# Uncemented three-dimensional-printed prosthetic reconstruction for massive bone defects of the proximal tibia

**DOI:** 10.1186/s12957-018-1333-6

**Published:** 2018-03-06

**Authors:** Minxun Lu, Yongjiang Li, Yi Luo, Wenli Zhang, Yong Zhou, Chongqi Tu

**Affiliations:** 1 Department of Orthopedics, West China Hospital, Sichuan University, No. 37 Guoxuexiang, Chengdu, 610041 People’s Republic of China; 2 Department of Oncology, West China Hospital, Sichuan University, Chengdu, People’s Republic of China

**Keywords:** Proximal tibia, Massive defects, Epiphysis preservation, 3D-printed prosthesis, Porous

## Abstract

**Background:**

Currently, it is challenging to treat massive bone defects of proximal tibia. Although numerous methods are available for reconstruction with epiphysis preservation, limitations in knee function and complications are noted with these methods. Our paper describes our attempt to reconstruct a marked defect in the proximal tibia with an uncemented three-dimensional (3D)-printed prosthesis and to evaluate the prosthesis design and short-term outcomes.

**Case presentation:**

A 15-year-old boy with metaphyseal osteosarcoma of the tibia underwent intercalary allograft reconstruction following wide tumour resection with epiphysis preservation. However, chronic allograft rejection and/or infection occurred after the surgery and a sinus tract was formed. The rejection and/or infection process was successfully stopped by the removal of the graft and implantation of an antibiotic-loaded cement spacer; however, the limb function was poor. Because of the irregular shape of the defect and the excessively short length of the residual proximal tibia, we used the 3D printing technology to design and fabricate a personalised prosthesis to reconstruct the defect, with the preservation of the knee joint. At the last follow-up at 26 months, the patient had satisfactory limb function.

**Conclusions:**

The 3D-printed prosthesis may be a feasible option in the reconstruction of tibial metaphyseal defects with the preservation of the knee joint. Moreover, it can result in good postoperative function and low complication rates. However, a long-term follow-up is required to clarify its long-term outcomes.

**Electronic supplementary material:**

The online version of this article (10.1186/s12957-018-1333-6) contains supplementary material, which is available to authorized users.

## Background

Primary malignant bone tumours mostly occur in the metaphysis of long bones [1, 2]. Although the growth plate is considered capable of preventing tumour spread, it is not impenetrable [3, 4]. Resection with epiphysis preservation is available for tumours that are not in contact with the growth plate (type I) [5]. However, if a tumour transgresses the epiphysis (type III) or is in contact with part or all of the epiphysis (type II), preservation of the epiphysis is contraindicated [5].

Several techniques have been applied for the reconstruction of proximal tibia defects following the resection of type I tumours, including distraction osteogenesis [6], intercalary allografting [4], fibular grafting [7], and prosthetic replacement [8]. Although these procedures have been reported to result in reasonable functional outcomes, they have their own limitations. The long duration of placement of the external osteosynthesis materials, the frequency of pin-tract infection, and the pain accompanying transport are common complications of distraction osteogenesis [6]. Graft transplantation has been reported to have a potential risk for nonunion, delayed union, and graft fracture [4, 7]. However, epiphysis-preserving reconstruction is usually difficult for the rigid fixation of the intercalary implant because of insufficient space of the residual bone [8].

We proposed that a custom-made uncemented three-dimensional (3D)-printed prosthesis, with a best-fit shape and a porous surface, would be a better choice for the reconstruction of a massive defect of the proximal tibia with preservation of the epiphysis. However, there is no related study regarding this approach. Our paper describes this 3D-printed prosthesis reconstruction in a patient with a challenging defect caused by osteosarcoma resection and destruction of the cement spacer.

## Case presentation

### History

**Fig. 1:**
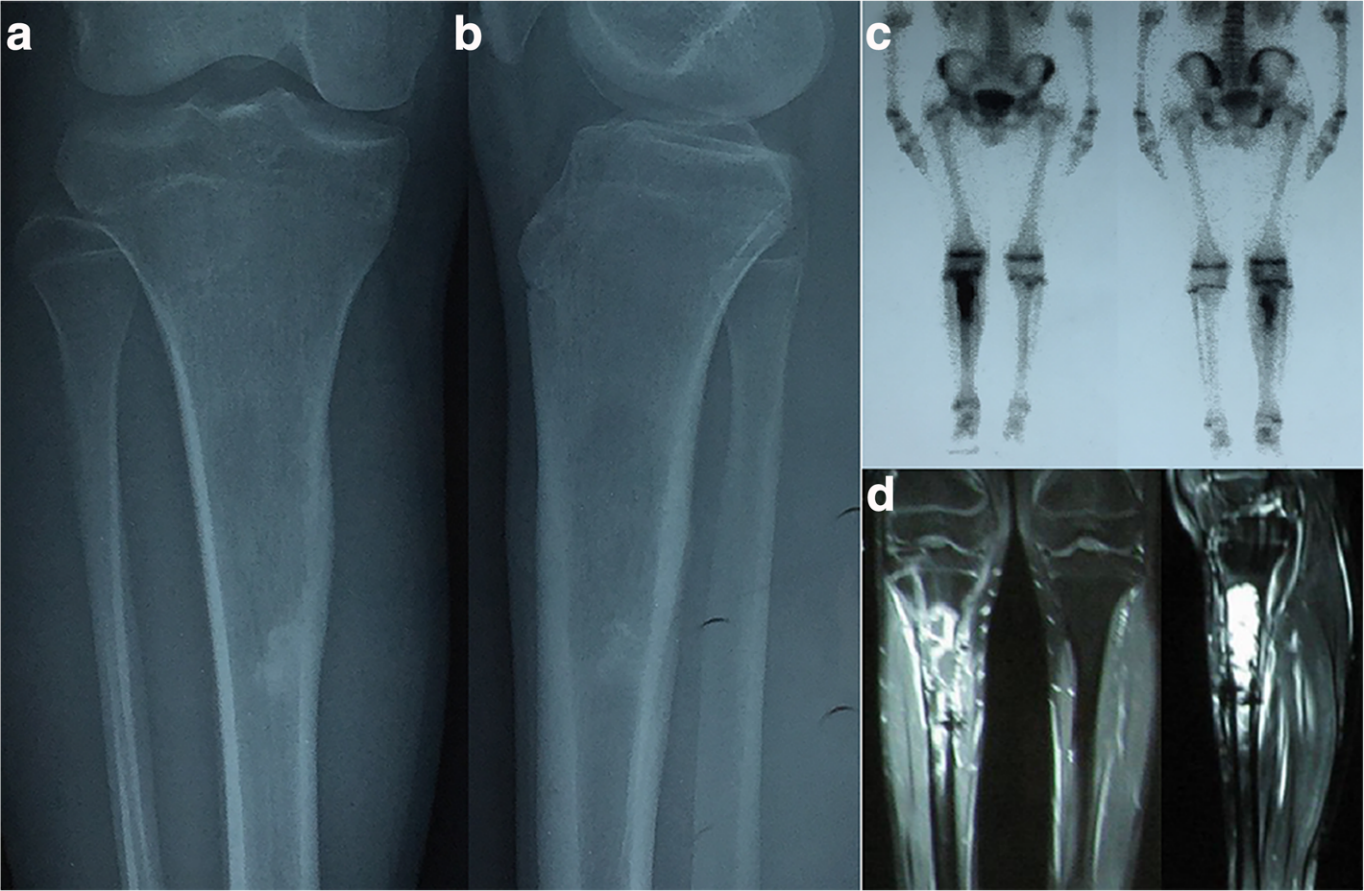
Anteroposterior (**a**) and lateral (**b**) radiographs, bone scan (**c**), and MRI (**d**) showing metaphyseal osteosarcoma of the right proximal tibia

**Fig. 2:**
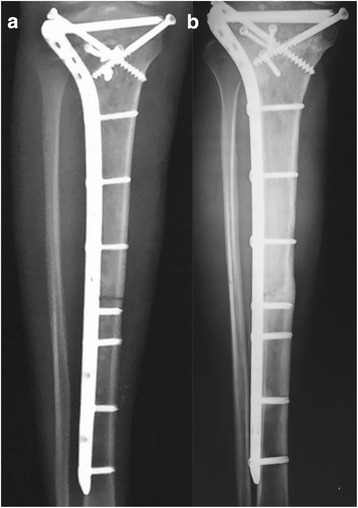
**a** Anteroposterior radiographs of right tibia after osteosarcoma resection and intercalary allograft reconstruction. **b** Anteroposterior radiographs of right tibia 21 months after the initial surgery

A 15-year-old boy complained of progressive pain in the right knee region for 3 months. He visited our institution and underwent a radiography examination in March 2011. The radiographs showed an expandable osteolytic lesion in the metaphyseal part of the tibia (Fig. 1a). After examinations including magnetic resonance imaging, computed tomography (CT), and bone scan (Fig. 1c), an incisional biopsy was performed. Our substantive diagnosis was osteosarcoma. After four courses of preoperative chemotherapy, segmental resection was performed 3 cm away from the tumour border. The epiphysis of the proximal tibia was successfully preserved. Thereafter, the tumourous defect was reconstructed with a frozen intercalary allograft and internal fixation (Fig. 2a). Postoperative chemotherapy was started 4 weeks after surgery.

**Fig. 3:**
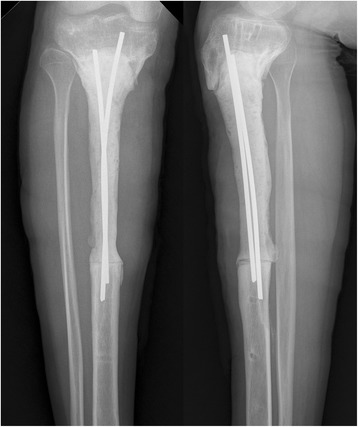
Anteroposterior and lateral radiographs of right tibia after removal of the intercalary allograft and reconstruction with temporary cemented spacer

Regular follow-up in the first year did not identify any clinical or radiographic evidence of recurrence or complications. However, 21 months after the primary surgery, the patient complained of pain in the previous surgical region; moreover, a sinus tract was noted. The erythrocyte sedimentation rate (ESR) and C-reactive protein (CRP) levels were twofold and fivefold the normal limits, respectively, but no bacterial growth was detected. No obvious bone deconstruction was found in X-ray (Fig. 2b). The patient was suspected to have a chronic allograft rejection and/or infection. To prevent the progression of the rejection process and possible implantation infection, the allograft and plate were removed and the segmental defect was reconstructed with an antibiotic-loaded cement spacer, containing 3 g of tobramycin and 1 g of vancomycin per 40-g package of bone-cement powder, and with two Kirschner wires (Fig. 3).

**Fig. 4:**
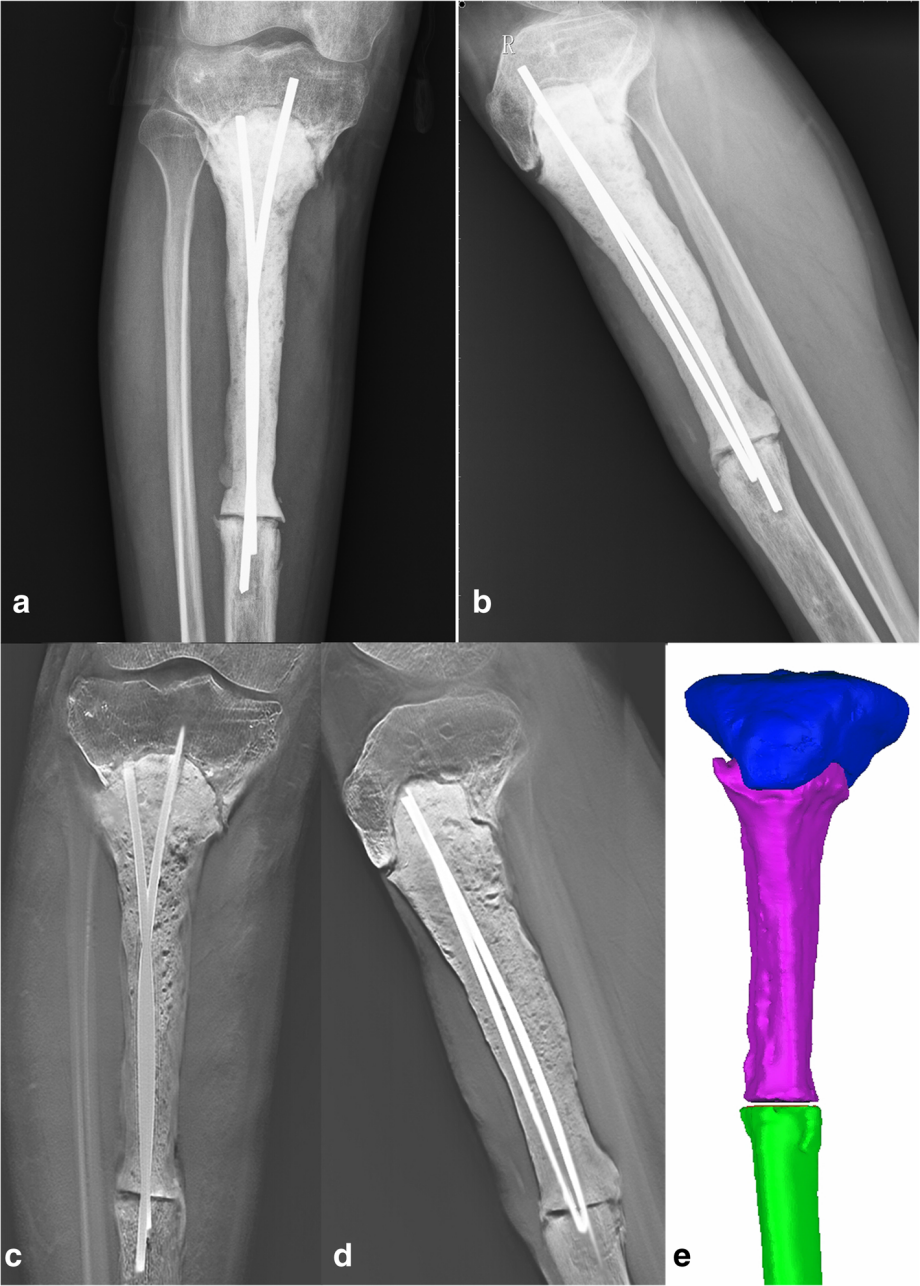
Anteroposterior (**a**) and lateral (**b**) radiographs of right tibia 9 months after temporary cemented spacer reconstruction. A distinct gap between the proximal tibia and the cemented spacer could be observed in anteroposterior (**c**) and lateral (**d**) T-SMART scanning. **e** Reconstructed 3D models of the proximal tibia, cemented spacer, and distal tibia

After the second surgery, liquid oozing out of sinus tract reduced and the sinus tract was healed. The ESR and CRP levels normalised. However, the patient limped with intolerable pain due to the deconstruction of the tibia plate caused by friction between the spacer and bone 1 year after the second surgery. Radiographs showed the severe damage of the proximal tibia and a gap between residual bone and the spacer (Fig. 4). Thereafter, a CT scan revealed that the bone defect involved the metaphysis of the tibia and was markedly irregular. We then decided to reconstruct this irregular defect with a custom-made uncemented 3D-printed prosthesis rather than with a standard intercalary prosthesis, allograft, or autograft.

### Prosthesis design and fabrication

**Fig. 5:**
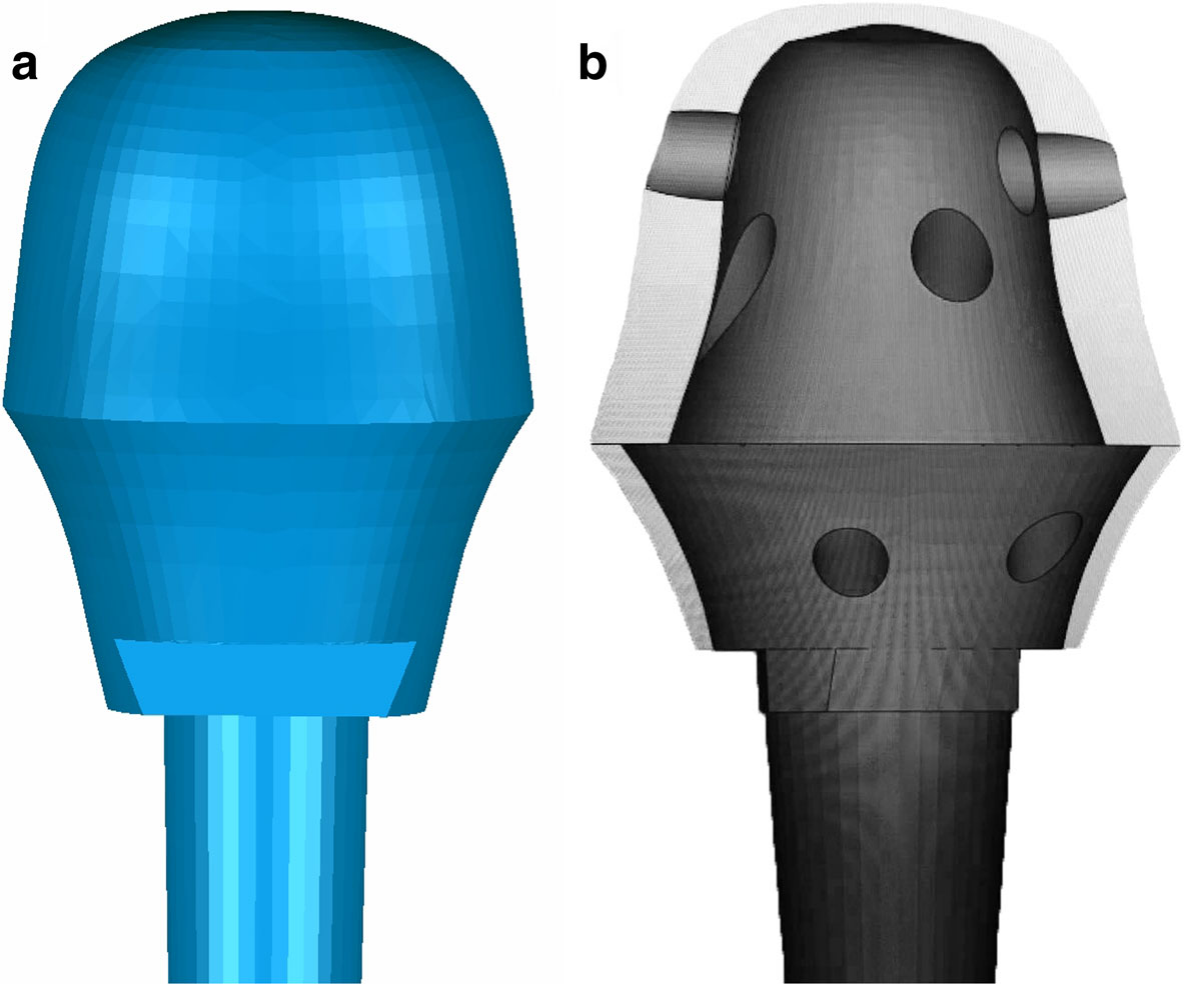
**a** Initial design of 3D-printed prosthesis. **b** Cross section of the initial design of 3D-printed prosthesis showing the outer-layer porous structure

The prosthesis was designed by our clinical team and fabricated by Chunli Co, Ltd., Tongzhou, Beijing, China. Improving the degree of matching between the prosthesis and bone defect was the major aim in our design process. Moreover, we considered enhancing the osteointegration capability of the prosthesis. For the first step, the 3D CT scan data was imported into the Mimics V17.0 software (Materialise Corp., Belgium). Building 3D computer models of the proximal tibia, the cement spacer, and the distal tibia was completed (Fig. 4e). The size of the proximal tibia defect, including the depth, width, and length of the defect, was precisely measured with the Mimics software. With this defect parameter, the initial prosthesis model was created, with a 27 mm height, 34 mm width, and 36 mm length, using Solidworks 2013 (Dassault Systemes, France). To ensure satisfactory fitting with the proximal tibia, the shape of the prosthesis was optimised via computer simulation (Fig. 5a). Six screw holes were designed to affix the proximal head of the prosthesis to the cortex of the tibia plate (Fig. 5b). The porous surface of the prosthesis head was designed to simulate trabecular structure with a porosity of 65%.

**Fig. 6:**
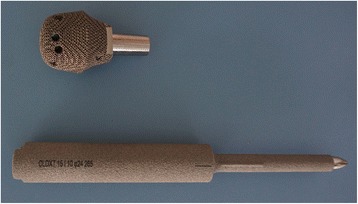
Final product of 3D-printed prosthesis

The proximal part of the prosthesis was fabricated by Electron Beam Melting technology (ARCAM Q10, Sweden). For the other parts of the prosthesis, traditional casting was applied to ensure mechanical strength. The stem of the distal part of the prosthesis was coated with hydroxyapatite (Fig. 6).

### Surgery

**Fig. 7:**
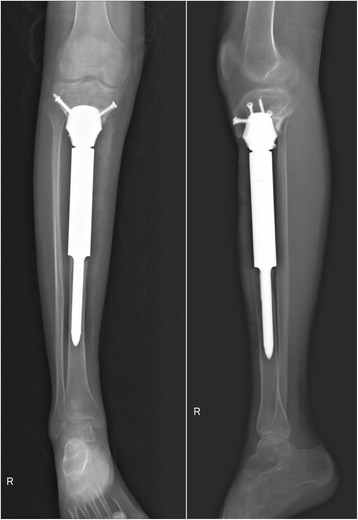
Anteroposterior and lateral radiographs after reconstruction with the 3D-printed prosthesis

The surgery was performed by the senior surgeon (Chongqi Tu). The cement spacer was removed, and the proximal tibia was reamed to fit the 3D-printed prosthesis. Implanting the proximal porous head of the prosthesis exactly at the preoperative position was relatively demanding. Moreover, the stem of the distal part was press fit inserted into the reamed distal tibia. After confirming both locations of the proximal and distal parts of the prosthesis, five screws were inserted to enhance primary fixation stability (Fig. 7).

### Postoperative management

Considering insufficient primary stability, the patient was allowed non-weight-bearing standing and walking with two crutches 3 weeks after surgery. Range of motion exercises of the knee were performed from postoperative week 4. Partial weight-bearing with crutches was encouraged from 6 weeks postoperatively, followed by gradual full weight-bearing. The patient was followed up every month for the first 3 months, then every 2 to 6 months to date. The range of motion, Enneking functional evaluation score, and radiographs were assessed at each follow-up visit to verify the outcomes of the 3D-printed prosthesis reconstruction.

### Outcome

**Fig. 8:**
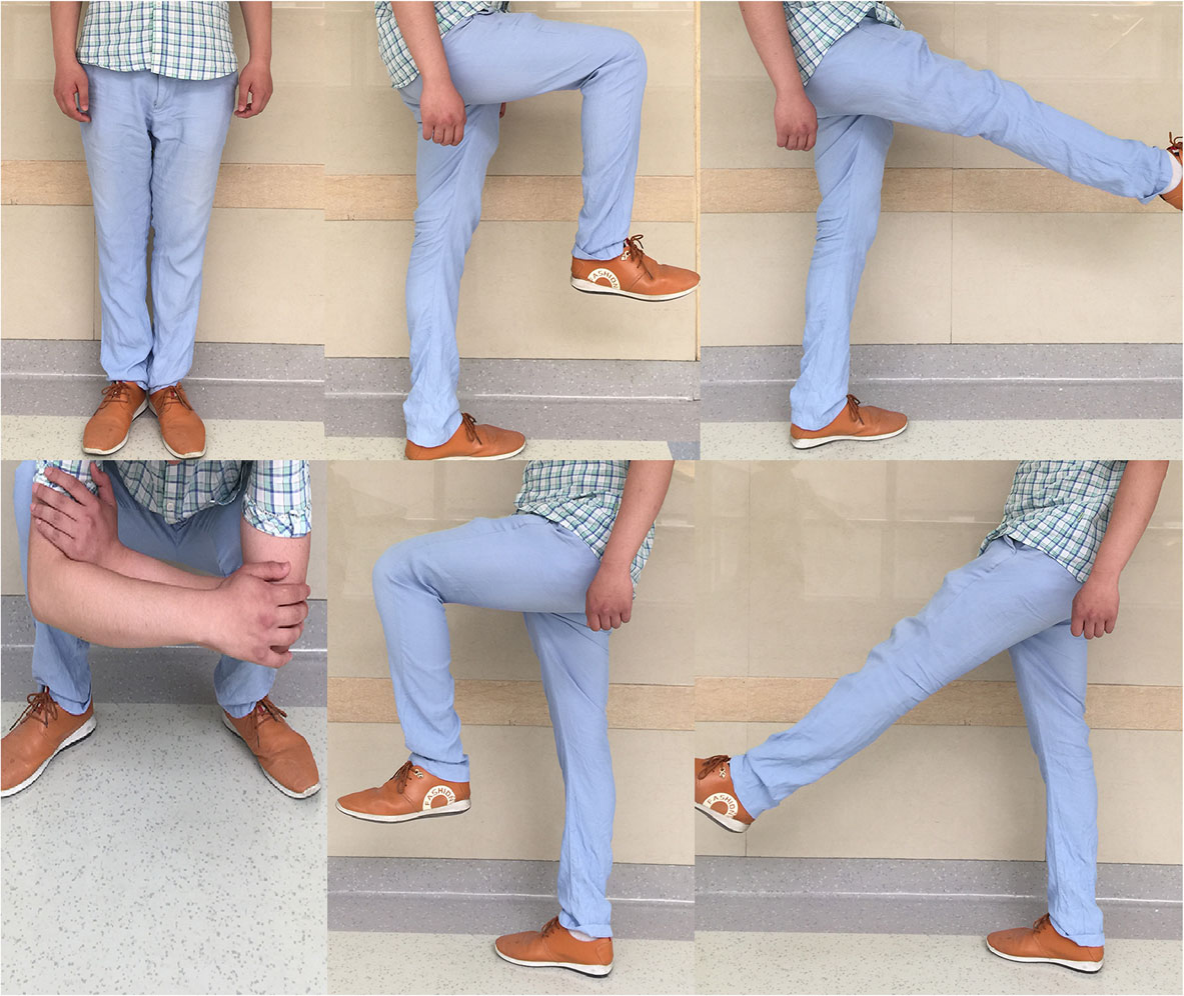
Satisfactory limb function 26 months after 3D-printed prosthesis reconstruction

**Fig. 9:**
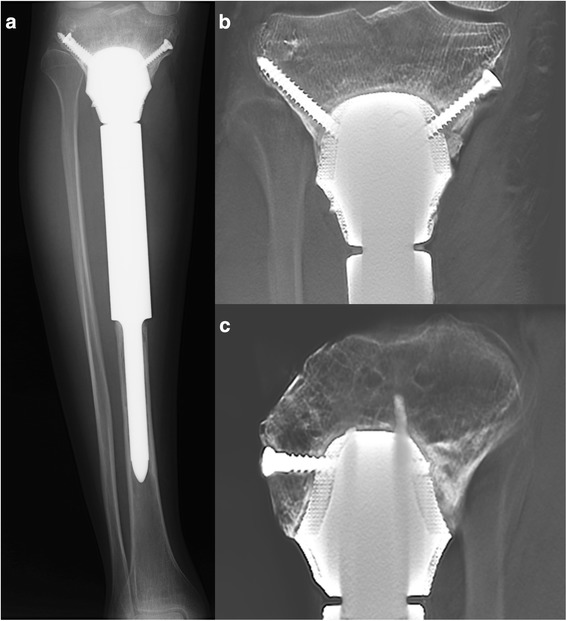
**a** Anteroposterior radiograph of right tibia 26 months after 3D-printed prosthesis reconstruction. Anteroposterior (**b**) and lateral (**c**) T-SMART scanning showed that the 3D prosthesis tightly integrated with the proximal tibia, and partial bone substance could be observed to grow into the porous structure of the prosthesis


At the last follow-up at 26 months, knee joint function was satisfactory (Fig. 8 and Additional file 1: Video), with a knee joint motion of 0°–130° and an Enneking functional evaluation score of 28 out of 30 (93.3%). Radiography indicated that the prosthesis fitted well with the tibia and no signs of complications were found (Fig. 9a). The Tomosynthesis-Shimadzu metal artefact reduction technology (T-SMART) scan showed that the 3D-printed prosthesis was well integrated with the proximal tibia (Fig. 9b, c).

Additional file 1:Satisfactory limb function. (AVI 4240 kb)


## Discussion

The proximal tibia is the second most frequent site for primary sarcomas in skeletally immature individuals. Furthermore, tumours occur more commonly near the metaphysis than diaphysis. According to the classification of San-Julian et al. [5], there is a chance of epiphysis preservation for only the type I lesion, where the tumour is isolated from the growth plate. In most cases, the proximal plane of the osteotomy is relatively close to the growth plate because of extended resection, meaning that the axial length of the residual proximal tibia is relatively short. Reconstruction of this kind of defect is considered challenging. However, various reconstruction procedures for segmental bone defects of the proximal tibia have been attempted in this region. Although distraction osteogenesis [6], intercalary allografting [4], fibular grafting [7], and prosthetic replacement [8] are acceptable techniques for this kind of reconstruction, an excessively short axial length of the remaining tibia, irregular defect shape, and the demand of weight-bearing could restrict the application of these common techniques. Considering the several advantages of the 3D-printed prosthesis, such as high shape compatibility, osteointegration capacity of the porous surface, and acceptable mechanical strength of the shaft and stem, we performed a reconstruction using a custom-made uncemented 3D-printed prosthesis in our patient. We found that the postoperative function was satisfactory and that no complications occurred.

In our case, we observed notable relief from pain and improvement in knee range of motion. Additionally, walking, running, and even jumping were possible for the patient after the surgery. No prosthesis-related complications were found. Previous studies have reported several reconstruction procedures after resection with epiphysis preservation. Although distraction osteogenesis is able to regenerate living bone of sufficient strength to support normal or near-normal function, deep infection resulting from long-term external fixation, long duration to full function restoration, fracture, deformity, nonunion, and delayed union are major complications [9]. In a study by Tsuchiya et al. [10], 31 patients with musculoskeletal tumours underwent this type of reconstruction from 1990 to 2002. After an average of 55.4 months of follow-up, complications occurred in 15 cases. Delayed consolidation, fracture, deep infection, pes equines, and skin invagination were noted in 2 patients each (6.5%), and nerve palsy, skin necrosis, deformity, premature consolidation, and fibular subluxation occurred in 1 patient each (3.2%). The mean external fixation time was 244 days, and it took 5 years for full function to be restored. Allografts have been widely used as an alternative choice for the reconstruction of proximal tibial resections, especially in cases where the tibial epiphysis can be preserved. Biological reconstruction is permanently achieved if the graft and host bone are well integrated. However, allograft-associated complications are relatively common, including graft rejection, nonunion, delayed union, bone absorption, and graft fracture. In addition, the size and shape of the graft hardly match with the defect of patients, and graft sources are limited. All these disadvantages of allografts increase the risk of failure of biological reconstruction. Weitao et al. [11] reported 5 cases of massive allograft transplantation in the proximal tibia after tumour resection with epiphysis preservation. The flexion and extension of the operated knee joint reached 128.67° and 1.80°, respectively. However, delayed bone healing in the allograft-host junction was noted in all cases. The vascularised fibular graft may decrease the rate of complications and achieve biologic reconstruction more easily, but it still has limitations such as a low degree of matching and inadequate strength to maintain satisfactory function. Intercalary prosthetic replacement is another therapeutic option for the reconstruction of the proximal tibia when the epiphysis can be preserved. Because of prosthesis stability produced by the intramedullary stem, an excessively short length of the residual tibia may not allow effective fixation, which means that a diaphyseal bone defect is a more appropriate indication than a metaphyseal bone defect. When the length of the remaining proximal tibia is longer than 4 cm, intercalary fixation with a short-segment intramedullary stem may be feasible [8]. Thus, it is challenging to reconstruct an extremely short proximal tibia using a prosthesis. Contrary to previous reports, we found that the postoperative flexion of the knee could reach 130°, while postoperative extension could reach 0°, which are relatively close to the normal range of knee joint motion. Compared to the previous gait of our patient, there was a notable improvement 12 weeks after the surgery. In addition, we observed no complications associated with the prosthesis, including loosening or breakage, at the final follow-up. Furthermore, the T-SMART scan revealed that the bone substance was well-wrapped around the porous surface of the prosthesis without any gap, indicating biological reconstruction.

Apart from the shape and porous surface of the proximal part of the prosthesis, a suitable length of the shaft, use of hydroxyapatite coating, and effective screw fixation would be the main factors that result in good function, with a low possibility of complications. First, the shape of the proximal part of the prosthesis is essential to achieve a high degree of matching and strong primary stability because of the limited contact area between the implant and the bone. To achieve this, the shape of the prosthesis was designed considering the shape of the defect, which means that the prosthesis was complementary to the proximal tibia defect. However, the initial shape we created was extremely irregular, which could have made it very difficult to satisfactorily couple with the proximal defect in actual surgery. Thereafter, we refined its design to make its shape regular for simpler implantation. Second, the porous structure imitating the trabecula could achieve osteointegration of the implant-bone interface [12]. Moreover, more friction force was generated to stabilise the interface because of the uneven porous surface. A well-integrated interface is difficult to break or loosen. Considering both the mechanical capacity and osteointegration ability of the proximal part, we chose the 600-μm size porous structure with 65% porosity [13].

The 3D-printed prosthesis may become mainstream for the reconstruction of bone defects caused by infection, tumour, or trauma. The reasons for this include that the personalised 3D-printed prosthesis, which is specifically tailored for the defect, may be easily designed and fabricated through 3D printing technology. Another reason is that the sophisticated porous structure of the prosthesis surface, produced by Electron Beam Melting technology, allows the easier formation of a well-integrated implant-bone interface compared to hydroxyapatite coating, with a low rate of complications. Moreover, 3D-printed production has sufficient mechanical strength to bear the body weight. Finally, the titanium alloy powder, which is a major material of 3D-printed implants, is more available than the allograft in most institutions.

With these advantages of 3D printing technology, several clinical applications of this technique have been reported. In the study by Wong et al. [14], they reconstructed the partial acetabular defects following pelvic chondrosarcoma resection with a computer-aided design implant fabricated by 3D printing technology. The patient could walk unaided with a good hip function 10 months after the surgery. No recurrence and implant loosening were noted at 11 months after surgery. In 2015, Fan et al. [15] reported about 3 patients with clavicular Ewing’s sarcoma, scapular Ewing’s sarcoma, and pelvic chondrosarcoma who underwent 3D-printed titanium prosthetic replacement. The prostheses were manufactured with a computer-aided design based on the anatomical shape of the affected bone. The Electron Beam Melting system was applied to manufacture the implants. With a mean duration of 21-month follow-up, the postoperative function was acceptable in each case. Moreover, the Musculoskeletal Tumour Society scores were 93, 73, and 90% for the patients with clavicle Ewing’s sarcoma, scapular Ewing’s sarcoma, and pelvic chondrosarcoma, respectively. No surgical complications including loosening and breakage were observed. Dai et al. [16] constructed a stereolithographic model of the pelvis to simulate bone resection and prosthesis installation. Thereafter, 10 patients underwent customised 3D-printed hemipelvic prostheses implantation following internal hemipelvectomy for extensive pelvic tumours. Excluding 4 patients that died of disease within 6 to 10 months postoperatively, the remaining 6 patients had good hip function after an average of 34.3-month follow-up.

We recognise the following limitations of this report. First, the follow-up duration was short. Thus, it is possible that additional complications or problems might arise as we follow up this patient for longer periods. However, our preliminary results seem reliable considering that most complications occur within 2 years after surgery. Second, one case is insufficient to verify the advantages of the 3D-printed prosthesis. Moreover, recruiting a large number of patients in one institution is difficult because, currently, standard indications for the 3D-printed prosthesis are few. The complicated approval process and the high cost of 3D printing are some of the reasons why this modality is not widely used. Therefore, a larger multi-institutional study is needed to adequately compare this approach with other types of reconstruction. Despite these shortcomings, this case study may provide valuable direction for further studies.

## Conclusions

This case report suggests that 3D-printed prosthetic replacement may be a feasible therapeutic option for the reconstruction of proximal tibia defects with epiphysis preservation. The well-fitting shape of the prosthesis, the integration ability of the porous structure, and the weight-bearing capacity of the shaft could lead to satisfactory knee function and a low rate of complications. However, as we have presented only short-term follow-up outcomes, the long-term efficacy of this technique regarding postoperative knee motion is yet to be clarified.

## References

[1] Zhang P, Feng F, Cai Q, Yao W, Gao S, Wang J, Wang X (2014). Effects of metaphyseal bone tumor removal with preservation of the epiphysis and knee arthroplasty. Exp Ther Med.

[2] Ottaviani G, Jaffe N (2009). The epidemiology of osteosarcoma. Cancer Treat Res.

[3] Betz M, Dumont CE, Fuchs B, Exner GU (2012). Physeal distraction for joint preservation in malignant metaphyseal bone tumors in children. Clin Orthop Relat Res.

[4] Muscolo DL, Ayerza MA, Aponte-Tinao LA, Ranalletta M (2005). Partial epiphyseal preservation and intercalary allograft reconstruction in high-grade metaphyseal osteosarcoma of the knee. J Bone Joint Surg Am.

[5] San-Julian M, Aquerreta JD, Benito A, Canadell J (1999). Indications for epiphyseal preservation in metaphyseal malignant bone tumors of children: relationship between image methods and histological findings. J Pediatr Orthop.

[6] Xu SF, Yu XC, Xu M, Chen X (2014). Successful management of a childhood osteosarcoma with epiphysiolysis and distraction osteogenesis. Curr Oncol.

[7] Manfrini M, Gasbarrini A, Malaguti C, Ceruso M, Innocenti M, Bini S, Capanna R, Campanacci M. Intraepiphyseal resection of the proximal tibia and its impact on lower limb growth. Clin Orthop Relat Res. 1999;358:111–9. 9973982

[8] Sewell MD, Hanna SA, McGrath A, Aston WJ, Blunn GW, Pollock RC, Skinner JA, Cannon SR, Briggs TW (2011). Intercalary diaphyseal endoprosthetic reconstruction for malignant tibial bone tumours. J Bone Joint Surg Br.

[9] Shirai T, Tsuchiya H, Yamamoto N, Sakurakichi K, Karita M, Tomita K (2004). Successful management of complications from distraction osteogenesis after osteosarcoma resection: a case report. J Orthop Sci.

[10] Tsuchiya H, Abdel-Wanis M, Kitano S, Sakurakichi K, Yamashiro T, Tomita K (2002). The natural limb is best: joint preservation and reconstruction by distraction osteogenesis for high-grade juxta-articular osteosarcomas. Anticancer Res.

[11] Weitao Y, Qiqing C, Songtao G, Jiaqiang W (2012). Epiphysis preserving operations for the treatment of lower limb malignant bone tumors. Eur J Surg Oncol.

[12] Murphy CM, Haugh MG, O'Brien FJ (2010). The effect of mean pore size on cell attachment, proliferation and migration in collagen-glycosaminoglycan scaffolds for bone tissue engineering. Biomaterials.

[13] Taniguchi N, Fujibayashi S, Takemoto M, Sasaki K, Otsuki B, Nakamura T, Matsushita T, Kokubo T, Matsuda S (2016). Effect of pore size on bone ingrowth into porous titanium implants fabricated by additive manufacturing: an in vivo experiment. Mater Sci Eng C Mater Biol Appl.

[14] Wong KC, Kumta SM, Geel NV, Demol J (2015). One-step reconstruction with a 3D-printed, biomechanically evaluated custom implant after complex pelvic tumor resection. Comput Aided Surg.

[15] Fan H, Fu J, Li X, Pei Y, Li X, Pei G, Guo Z (2015). Implantation of customized 3-D printed titanium prosthesis in limb salvage surgery: a case series and review of the literature. World J Surg Oncol.

[16] Dai KR, Yan MN, Zhu ZA, Sun YH (2007). Computer-aided custom-made hemipelvic prosthesis used in extensive pelvic lesions. J Arthroplast.

